# Developing Healthcare Complaints Analysis Tool for Health System: A Cross‐Sectional Study

**DOI:** 10.1002/hsr2.70325

**Published:** 2025-01-12

**Authors:** Ali Vafaee Najar, Damoon Gorji, Hoorang Nazari Ardabili, Maryam Pourshirazi, Elaheh Houshmand

**Affiliations:** ^1^ Social Determinates of Health Research Center Mashhad University of Medical Sciences Mashhad Iran; ^2^ Health Services Management Social Security Organization Semnan Iran; ^3^ Master Student in Health Services Management, Student research committee Mashhad University of Medical Sciences Mashhad Iran; ^4^ Department of Midwifery, School of Nursing and Midwifery Mashhad University of Medical Sciences Mashhad Iran; ^5^ Department of Health Management Sciences and Health Economics, School of Health Mashhad University of Medical Sciences Mashhad Iran

**Keywords:** HCAT, healthcare complaints, patients, tool

## Abstract

**Background and Aims:**

The role of the healthcare system in the provision, maintenance, and promotion of public health is associated with handling healthcare complaints. This notion as the principle of accountability requires the authorities' attention. This study aimed to develop the Healthcare Complaints Analysis Tool (HCAT) in Iran.

**Methods:**

Following a comprehensive review of the country and field studies conducted in the Medical System Organization and Universities of Medical Sciences, the features of patients' complaints were extracted from medical centers and hospitals. Then, experts who were experienced in health and medical areas localized HCAT through translation and re‐translation process using the Delphi technique. Finally, the healthcare complaints were classified according to the localized HCAT components.

**Result:**

Using HCAT showed that in Iran's healthcare system, complaints were related to relative frequency and percentage of all types of complaints. However, there is a lack of a mechanized registration system, investigation, analysis, and classification of healthcare complaints.

**Conclusions:**

Providing medical centers with such a system by the ministry of health and mandatory reporting of complaints may lead to coding complaints, measure the severity of complaints, service monitoring and organizational learning.

## 1 Introduction

Hospital is the frontline in providing specialized services and is important worldwide [1]. According to the role of the healthcare system in the provision, maintenance, and promotion of public health, handling healthcare complaints as the principle of accountability requires authorities' attention [2]. A complaint is a tool for expressing discontent and needs to be responded to. Healthcare complaints conventionally refer to an expression of grievance and dispute, typically written and communicated through a letter by a patient or their family, about the receipt of healthcare [3]. such as treatment delays, difficulty accessing treatment or delays in diagnosis [4].

The increase in the level of education in the community and the role of patients and their families in the management of the disease may lead to the increasing demand for high‐quality healthcare services and consequently more complaints [5]. Lack of information on patients' medical history, insufficient medical knowledge, and high workload could all play a part [6].

Healthcare complaint is a source for promoting the quality of healthcare services [7]. Several studies showed that analyzing healthcare complaints is beneficial to evaluating the safety and quality of care [8, 9]. Patients as service recipients can better understand the continuity of care and the related defects of providing care [10] Thus, they may evaluate the quality of service as an external evaluator independently [11]. Using complaints to access patient insights into safety and quality issues in general practice could provide valuable learning, given the frequency of contact and the privileged viewpoint that patients have within the health care system [4]. However, the tools to exploit the potential of this information are limited. Analyzing healthcare complaints is a beneficial method to evaluate the safety and quality of care [12].

The Healthcare Complaints Analysis Tool (HCAT) is the first standard tool for classification and analyzing healthcare complaints precisely and comprehensively [13]. The healthcare complaint analysis tool (HCAT) was defined by Gillespie et al., (2015) by aggregating the coding taxonomies from studies included in the systematic review, revealing 729 uniquely worded codes that were then refined and conceptualized into three broad domains and seven categories [13]. The HCAT taxonomy represents, the inaugural tool that is grounded in a comprehensive literature review and constructed through a meticulous and transparent methodology. The HCAT has been utilized across various countries and has proven effective in pinpointing ‘blind spots’ within healthcare systems. It provides a reliable tool for coding complaints and measuring the severity of complaints, to facilitate service monitoring and organizational learning [14].

HCAT increases the organizations' attention to medical complaints. Patients' opinions facilitate monitoring healthcare services and organizational learning. This tool classifies healthcare complaints into three disciplines including management, clinical, and communication. Each discipline consists of several groups and sub‐groups. There is a severity definition for each complaint group. HCAT is responsible for classifying the complaints based on the total severity rate and determining their priority [15]. According to the medical system organization report in Iran, there is a 5%–10% increase in the rate of healthcare complaints. However, according to the studies conducted in the country, there is no tool for separating, ranking, and analyzing healthcare complaints [16, 17, 18]. There is a lack of a formal taxonomy for the classification of healthcare complaints. Additionally, the procedures involved, such as coding guidelines and training, exhibit minimal standardization, and no HCAT has undergone comprehensive reliability testing. Furthermore, the analysis of healthcare complaints frequently prioritizes primary issues while neglecting secondary concerns. Lastly, although the severity of the issues presented can vary significantly—from minor grievances like parking fees to serious cases of medical negligence—current tools fail to evaluate the severity of these complaints [19].

Therefore, the present study aimed to design and implement a local model similar to HCAT to analyze healthcare complaints based on global standards. The application of this tool leads to several results and advantages such as promoting the safety and quality of health care, an increase in patient satisfaction, and efficiency and efficacy of health indices, and can be used as a guide for countries that have similar problems and conditions as Iran.

## 2 Methods

This cross‐sectional study was approved by Ethics Committee of Mashhad University of Medical Sciences (IR. MUMS. REC.1395.282), conducted in October 2021 and April 2022 to determine the reliability of the HCAT through the following steps:

Stage 1: Determining the features of complaints in Iran by the comprehensive review method.

We searched for all the studies and articles in the healthcare complaints area using a comprehensive review method. Information resources included an interview with the staff working in the unit of handling healthcare complaints in hospitals and conducting field studies in settings of Medical System Organization, Forensic Medicine Organization, and Mashhad, Tehran, Isfahan, Shiraz, and Semnan Universities of Medical Sciences. Electronic searches in databases, contacting authors by email, and reviewing scientific journals were done according to the study protocol to collect and retrieve papers and studies.

Keywords included “complaint,” “discontent,” “patient,” “healthcare,” “hospital,” and “analysis tool.” Information was extracted after removing plagiarism manually. Keyword searches in electronic databases, including SID, Iranmedex, Magiran and Irandoc, Medline, Scopus, EBSCO, PubMed, BMJ, and Science Direct were done. Then, we completed the information manually by searching related websites and contacting experts. Having evaluated the extracted articles by reading the title and abstract, the selected articles were then identified.

Stage 2: Localizing the HCAT [20].

Phase 1: Translation of the tool:

We used the translation and re‐translation method for phase 1 to make the tool adjusted to the culture of Iran. First, 2 translation experts translated the original version of the guideline for coding complaints using HCAT into the Persian language via the standard method the translators worked independently of each other. Then, 2 translators who were experts in the medical and healthcare field, again, translated the translated version into the English language, To ensure that the translation is done correctly

To make the questions more comprehensive and matched with the sociocultural situation of the country, most of them were revised. Finally, an expert in the medical and healthcare field, as a coordinator, prepared the guidelines for coding complaints using HCAT by matching the Persian and revised English versions with the original one.

Phase 2: Determining the Validity of the tool:

Step 1: The purpose was to validate the questionnaire by the application of the Delphi method. We selected 35 experts via the targeted sampling method according to the research team's opinions and similar studies were selected from the national level. The inclusion criteria were as follows:

An experienced faculty member of Health Services Management from Medical universities who had similar publications.

The head and managers of the Ministry of Health who had at least 5 years of work experience in public and private hospitals.

The manager of quality control in the public and private hospitals of Medical Universities.

Step 2: After all participants gave informed consent for participation in the study, they filled out the questionnaire either in person or via e‐mail. In case of misunderstanding about the concepts of the questions, they were asked to write down their comments and corrective feedbacks in the questionnaire. At last, the questionnaires were collected and analyzed. We applied a scoring system from zero to 100. If the score was below 60%, the corrective feedback was applied and the edited questions were then sent to the participant.

The score for the indices such as patient environment and respectful behavior was in the range of between 40% and 60%. The former indices were then evaluated by the application of the Delphi method and scored above 60% [21]. Moreover, factors such as security and mandatory feeding that scored below 40% were deleted according to the Delphi team.

The average score for participants' answers to each component of the questionnaire was 85% and the standard deviation for their comments was estimated to be 20%. Therefore, the research team reached a consensus on the comments using the 2‐step Delphi method. Data were analyzed using SPSS.

Step 3: The healthcare complaints in Iran were classified according to the localized HCAT.

## 3 Results

Stage 1: Determining the features of complaints in Iran by the comprehensive review method.

**Figure 1:**
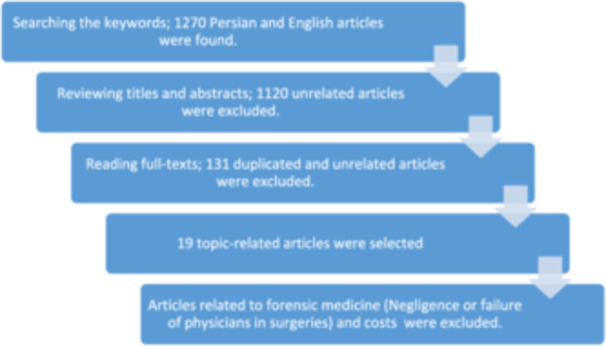
The process of reviewing and selecting the articles.

Figure 1 shows the process of reviewing and selecting the articles. Table 1 shows the features of the healthcare complaints in Iran in terms of the type of complaint.

**Table 1:** The features of healthcare complaints in Iran.

No.	The features of healthcare complaint in Iran
1	Quality of care
2	General issues such as cleanliness and hoteling
3	Disrespectful behavior of the Physicians
4	Disrespectful behavior of the medical and nonmedical staff
5	Unnecessary procedures or application of inappropriate methods
6	Delays in service delivery
7	Unauthorized or non‐standard medical centers
8	Medical complications
9	Physical injuries
10	Psychological injuries
11	Delayed or canceled meeting time of the patient
12	Inappropriate communications
13	Administrative and structural problems
14	Poor quality of service
15	Welfare facilities
16	Nutrition
17	Lack of appropriate explanation about the services
18	Wrong diagnosis
19	Lack of physical space
20	Lack of human resource
21	Not respecting patients confidentiality and privacy
22	Lack of diversity in specialties
23	Lack of appropriate medical equipment
24	Defective queuing
25	Compliance failure
26	The noisiness of the medical environment

A systematic review of the features of healthcare complaints in Iran showed that the majority of the studies in this field were conducted in Tehran between 2011 and 2020. Moreover, all the studies related to that period were done by universities and medical centers under the supervision of the Ministry of Health.

Based on the literature review, healthcare complaints in Iran were classified into 28 groups in terms of diversity. Two groups related to cost complaints were excluded from the study.

Stage 2: Localizing the HCAT.

Phase 1: Translation of the tool:

To make the questions comprehensive through the Delphi process, we made adjustments for some words and phrases in the translation and re‐translation phase. For instance, the word “HEALTHCARE” was replaced with the word “MEDICAL” to be more comprehensive for the experts in terms of cultural issues. The phrase “MAKING & FOLLOWING CARE PLANS” was changed to the phrase “COMPLIANCE WITH TREATMENT PROGRAMS”. According to the experts' opinion, the phrase “PROBLEM IN FACILITIES” was replaced with the phrase “PATIENT'S COMFORT”.

Phase 2: Determining the Validity of the tool:

Table 2 shows the demographic characteristics of the participants according to the Delphi method.

**Table 2:** Gender and level of education of experts participating in the Delphi process.

Level of education	Master's degree		PhD		Specialist physician		Total	
Gender	Frequency	Percent	Frequency	Percent	Frequency	Percent	Frequency	Percent
**Female**	12	80	1	7	2	13	15	43
**Male**	8	40	4	20	8	40	20	57
**Total**	20	57	5	14	10	29	35	100

According to the HCAT, healthcare complaints are categorized into three main groups: (1) clinical problems, (2) management problems, and (3) communication problems.

Clinical problems are classified into two sub‐groups: (1‐1) quality of medical care and (1‐2) patient safety. Based on the HCAT, quality of medical care includes personal healthcare, defects in patient's nutrition, public negligence, insufficient control and discomfort, monitoring and auditing the status, compliance with treatment programs, and side effects. Patient safety includes errors in diagnosis, errors in medication, general errors, defects in responding to the patient, specialist physician's skills, and defects in teamwork, From which patient's nutrition and public negligence had a higher level of agreement.

Management problems are classified into two sub‐groups: (2‐1) patient environment and (2‐2) defects in administrative and supportive processes, waiting time, and medical assessments. The patient environment includes the patient's comfort, preparation for patient bed space, cleanliness, equipment, sufficient staff, and patient mental comfort. Defects in administrative and supportive processes, waiting time, and medical assessments include delay in access to healthcare services, delay in intervention, general delays, defects at the time of consultation and appointments, and defects in the patient health profile documentation. From which waiting time and delay in access to healthcare services had a higher level of agreement.

Communication problems are classified into two sub‐groups: (3‐1) listening to the patients and their concerns, (3‐2) communication, and (3‐3) patients' respect and rights. Listening to the patients and their concerns includes insufficient attention to the patient, failure to care for the patient, and lack of attention to the nonmedical requests of patients. Communication issues include delays in communicating with the patient, keeping patients' secrets and information, respect for patients' rights and equality, obtaining the patient's consent for medical treatment, and patients' privacy. From which keeping patients' secrets and information and patients' privacy had a higher level of agreement.

Based on the mentioned classification, coding a healthcare complaint using HCAT consists of four stages (A–D). Table 3 describes the whole process.

**Table 3:** The process of coding a healthcare complaint using HCAT.

Stage	Description
A	Identifying the problem classification (and subcategories, if needed) in the complaint letter using coding categorization and assessing their severity (clinical, management, and communication problems defined as severe, moderate, and mild in terms of severity).
B	Determining the medical stages in which the problem occurred (admission, physical examination, and diagnosis, treatment, surgery, discharge, transfer to another facility, etc).
C	Display the level of damage caused by the reported problem (Minimal damage, low damage, moderate damage, severe damage, catastrophic damage)
D	Providing descriptive information about the complaint letter (Who has complained? What is the patient's gender? What group of employees does the complaint refer to?)

Step 3: The Healthcare complaints in Iran were analyzed according to the localized HCAT. Table 4 shows the classification of the features of healthcare complaints in Iran according to the HCAT.

**Table 4:** The features of healthcare complaints in Iran according to the HCAT.

No.	The feature of healthcare complaint	The classification of the features of healthcare complaints according to the HCAT
1	Quality of care	Quality of care, patient safety
2	General issues such as cleanliness and hoteling	Patient's environment
3	Disrespectful behavior of the Physicians	Communication, listening, and respecting patients' rights
4	Disrespectful behavior of the medical and nonmedical staff	Communication, listening, and respecting patients' rights
5	Unnecessary procedures or application of inappropriate methods	Quality of care, patient safety
6	Delays in service delivery	Quality of care, administrative support services, waiting time, and evaluation of treatment by the patient
7	Unauthorized or nonstandard medical centers	Quality of care, patient safety
8	Medical complications	Quality of care, patient safety
9	Physical injuries	Quality of care, patient safety
10	Psychological injuries	Behavior and respecting patients' rights
11	Delayed or canceled meeting time of the patient	Administrative support services, waiting time, and evaluation of treatment by the patient
12	Inappropriate communications	Communication, listening
13	Administrative and structural problems	Administrative support services, waiting time, and evaluation of treatment by the patient
14	Poor quality of service	Quality of care, patient safety
15	Welfare facilities	Patient's environment
16	Nutrition	Quality of care
17	Lack of appropriate explanation about the services	Listening
18	Wrong diagnosis	Quality of care, patient safety
19	Lack of physical space	Patient's environment
20	Lack of human resource	Patient's environment
21	Not respecting patients confidentiality and privacy	Respect patients' rights
22	Lack of diversity in specialties	Administrative support services, waiting time, and evaluation of treatment by the patient
23	Lack of appropriate medical equipment	Patient's environment
24	Defective queuing	Administrative support services, waiting time, and evaluation of treatment by the patient
25	Compliance failure	Respect patients' rights
26	The noisiness of the medical environment	Patient's environment

## 4 Discussion

This cross‐sectional study aimed to presents the development and evaluation of a tool designed for the analysis of healthcare complaints in Iran.

Comparing the local model of HCAT is designed in this study with the original HCAT shows that according to the opinion of the researchers who participated in the Delphi rounds, due to the cultural difference between Iran and England, changes were made in the vocabulary or the structure of the questionnaire, which were finally approved by the experts.

So, like its original model it consists of three main domains: (1) clinical problems, (2) management problems, and (3) communication problems which divided to Seven main group and 36 subgroups.

This article demonstrates that HCAT is proficient in accurately identifying issues, assessing their severity, determining the stage of care, and recognizing harm as reported in healthcare complaints. This tool plays a significant role in the three domains it evaluates. Wray and colleague in their study entitled “Testing the Healthcare Complaints Analysis Tool in a Specialist Pediatric Hospital to Assess Potential Utility for Organizational Learning from Complaints” indicated that, although complaints received by their organization are complex compared with many other organizations, using the HCAT scoring taxonomy was feasible [22].

O'Dowd et al reports the adaptation of the HCAT for general practice and has established that the tool has sufficient validity, reliability and usability. they believed that This adapted tool can be applied to general practice complaints to identify areas for improvement [23].

Jerng in a study titled “Comparison of complaints to the intensive are units and those to the general wards: an analysis using the Healthcare Complaint Analysis Tool in an academic medical center in Taiwan” states that A structured typing and systematic analysis of the healthcare complaints to the ICUs may provide valuable insights into the improvement of care quality, especially to the perceptions of the ICU environment and communications of the patients and their families [15].

Bie Bogh et al In their study, conclude that HCAT was found to be a reliable tool for categorizing problem types in patient compensation claims [14].

The results of our study also shows that HCAT has great potential to support the use of patient complaints for safety and quality, it provides a reliable means through which complaints managers can analyses patient complaints.

According to the localized HCAT, 19 out of 26 cases (almost 75%) of the healthcare complaints were related to clinical and management issues. However, the review study by our research team showed that only 40% of the healthcare complaints were related to clinical and management problems. This controversy confirms that healthcare complaints are not analyzed and classified by the correct tool and the proper pattern [5].

Furthermore, HCAT plays a significant role in elucidating the relational aspects of patient experience. Approximately one‐third of complaints in healthcare pertain to the relational domain, and gaining deeper insights into these issues, as well as their connections to clinical and management practices, is crucial for enhancing patient satisfaction and perceptions of healthcare services.

Given that the HCAT includes various complaint classifications and also due to the lack of a legal obligation to force medical centers to register complaints, a mechanized registration system, and a tool to analyze and classify healthcare complaints as well as problems with researching by the health system authorities, especially for the private sector, the need for a tool to classify and analyze the healthcare complaints in Iran is quite noticeable.

HCAT has the potential to enhance healthcare management practices. Specifically, it could be incorporated into current complaint coding systems, enabling the extraction of HCAT severity ratings for dissemination to managers, external evaluators, and researchers. Furthermore, HCAT could serve as an alternative measure of success in achieving various standards, such as hospital cleanliness, patient wait times, and overall patient satisfaction [24]. Its longitudinal application could facilitate the evaluation of clinical, managerial, or relational interventions. Moreover, HCAT could function as a benchmarking tool for different units or regions. The collection of normative data would enable healthcare organizations to be assessed for discrepancies, whether they manifest as subpar or exemplary complaint profiles [13]. More than 90% of the healthcare complaints in Iran are related to disrespectful behavior and poor quality of care. The study by Mirzaaghae et al. showed that the majority of the healthcare complaints in Tehran hospitals are associated with disrespectful behavior and poor quality of care [25]. According to the modeling of the country's healthcare complaints from Tehran city, the main problem in providing a high quality of care to patients and their companions is the lack of communication and behavioral skills among medical staff. It is recommended that the Ministry of Health takes action to solve the problem by making regulations for medical staff to participate in communication skill courses.

Similar studies in the United Kingdom, the United States, Canada, and Australia reported that the majority of healthcare complaints are related to disrespectful behavior and poor quality of care [25, 26, 27].

To encourage state and private medical centers to register complaints, it is recommended to define specified criteria in the National Accreditation Booklet for registering and analyzing complaints and cultivate a trusted reporting culture.

Given that, the HCAT classifies the healthcare complaints into different domains, it is possible to refer complaints to the relevant units. This will avoid working in parallel and wasting time and money [28]. Moreover, it is possible to identify the stage and the time of the given care in which the complaint has occurred. Therefore, we can audit the patients' problems timely, effectively, and efficiently, with the least cost and most benefit to the patient with a retrospective or cross‐sectional view [23]. A significant advancement introduced by HCAT is its capacity to accurately assess the severity of complaints within each designated category. Historically, the examination of healthcare complaints has primarily focused on the frequency of issues reported, without consideration for their severity. This approach inadvertently disadvantages organizations that encourage feedback to enhance quality; it is plausible that an ideal complaint profile consists of a substantial proportion of low‐severity complaints, indicating that the institution not only welcomes feedback but also effectively mitigates the risk of serious failures.

Considering the evidence and reported efficiency and effectiveness of HCAT and its capability in classifying the features of health complaints in Iran, the tool is highly recommended to be used by the health authorities and policymakers to gain an efficient and effective scientific and practical vision for analyzing complaints and formulating related policies.

The limitation of the current study was the spatial and temporal restrictions on access to specialist physicians for consultation and completion of the Delphi form. This problem was resolved through arrangements made with their office secretary before making an appointment. Furthermore, a lack of information on the HCAT and a similar method in the country to classify and analyze complaints challenged the comparison of the results of this study with similar studies. Also, in this study, only the complaints registered in the field studies and the cases mentioned in the published articles were investigated and classified.

The strengths of this study are this article demonstrates that HCAT is an effective tool for accurately identifying issues, their severity, the stage of care, and the harm associated with healthcare complaints. It plays a significant role in monitoring three key domains. HCAT offers a consistent supplementary data source for assessing healthcare safety and quality. Furthermore, HCAT aids in comprehending the relational aspects of patient experiences. Approximately one‐third of healthcare complaints pertain to the relational domain, and gaining insights into these issues, as well as their connections to clinical and management practices, is crucial for enhancing patient satisfaction and perceptions of healthcare services. These more nuanced aspects of care have been challenging to monitor, yet HCAT can serve as a reliable additional data source. Lastly, HCAT can enhance healthcare management by being integrated into current complaint coding systems, allowing for the extraction of HCAT severity ratings to be communicated to managers, external monitors, and researchers.

## 5 Conclusion

The HCAT plays a significant role in healthcare management by facilitating the integration of existing patient complaints into its severity ratings. These ratings can subsequently be communicated to managers, external evaluators, and researchers. The HCAT serves as an alternative measure of success in achieving healthcare standards. Additionally, it can be employed to monitor the effectiveness of clinical, managerial, or relational interventions. By utilizing the HCAT, hospitals can gain insights into patient needs and expectations through their complaints, enhance the delivery of compassionate medical and health services, mitigate disparities in information dissemination, foster trust and quality within the doctor‐patient relationship, and ultimately improve patient safety and satisfaction.

## 6 Recommendation for Policy, Practice and Research

The HCAT (GP) enables researchers, practitioners, and policymakers to pinpoint the most prevalent issues within health care environments, thereby establishing a solid evidence base for the enactment of quality and safety improvement initiatives. The HCAT allows researchers and practitioners alike to categorize complaints based on their content, severity and harm caused to patients Furthermore, this tool can be applied at various levels of the healthcare system; although individual practices may report a limited number of complaints, the tool can initially categorize these complaints locally, with the aggregated data subsequently shared at regional or national levels to enable a more extensive analysis of complaint trends. The standardization of complaint categorization through this tool would bolster such higher‐level analyses, positioning it as both a categorization instrument and a foundational step in the examination of trends in healthcare complaints.

## Author Contributions

Study conceptualization and supervision: Ali Vafaee Najar and Elaheh Houshmand. Writing original draft: Damoon Gorji. Formal analysis and Investigation: Hoorang Nazari Ardabili. Writing (editing): Maryam Pourshirazi. Project administration: Elaheh Houshmand.

All authors have read and approved the final version of the manuscript (Elaheh Houshmand) had full access to all of the data in this study and takes complete responsibility for the integrity of the data and the accuracy of the data analysis.

## Ethics Statement

This research is approved by Ethics Committee of Mashhad University of Medical Sciences (IR. MUMS. REC.1395.282).

## Conflicts of Interest

The authors declare no conflicts of interest.

## Data Availability

The authors confirm that the data supporting the findings of this study are available within the article and its supplementary materials. Additional datasets used and analyzed during the current study are available from the corresponding author on reasonable request.
